# Amyloid-Beta Peptide, Oxidative Stress and Inflammation in Alzheimer's Disease: Potential Neuroprotective Effects of Omega-3 Polyunsaturated Fatty Acids

**DOI:** 10.4061/2010/274128

**Published:** 2010-06-14

**Authors:** S. C. Dyall

**Affiliations:** British College of Osteopathic Medicine, Lief House, 120-122 Finchely Road, London NW5 5HR, UK

## Abstract

Alzheimer's disease is the most common form of dementia in the elderly and is a progressive neurodegenerative disorder characterised by a decline in cognitive function and also profound alterations in mood and behaviour. The pathology of the disease is characterised by the presence of extracellular amyloid peptide deposits and intracellular neurofibrillary tangles in the brain. Although many hypotheses have been put forward for the aetiology of the disease, increased inflammation and oxidative stress appear key to be features contributing to the pathology. The omega-3 polyunsaturated fats, eicosapentaenoic acid (EPA), and docosahexaenoic acid (DHA) have well-characterised effects on inflammation and may have neuroprotective effects in a number of neurodegenerative conditions including Alzheimer's disease. The aims of this paper are to review the neuroprotective effects of EPA and DHA in Alzheimer's disease, with special emphasis on their role in modulating oxidative stress and inflammation and also examine their potential as therapeutic agents.

## 1. Introduction

Alzheimer's disease (AD) is the most common form of dementia in the elderly. It is a progressive neurodegenerative disorder characterised by a decline in cognitive function and also profound alterations in mood and behaviour [1]. The pathology of the disease is characterised by the presence of extracellular amyloid peptide deposits, soluble amyloid *β*-protein and hyperphosphorylated tau protein leading to the formation of intracellular neurofibrillary tangles in the brain. The aetiology and pathogenesis of the disease are currently poorly understood, and the present management is to a large extent symptomatic and focused on ameliorating the cognitive deficits [2]. However, although many hypotheses have been put forward for the aetiology of the disease, increased inflammation [3] and oxidative stress [4] appear to be key features contributing to the pathology of the disease. 

The omega-3 polyunsaturated fatty acids (PUFA), eicosapentaenoic acid (EPA), and docosahexaenoic acid (DHA) have well-characterised effects on inflammation [5] and may have neuroprotective effects in a number of neurodegenerative conditions including AD [6]. The purpose of this paper is to review the neuroprotective effects of EPA and DHA in AD with emphasis on their potential for modulating amyloidosis and the increased oxidative stress and inflammation seen with AD and explore their potential application as therapeutic agents.

## 2. Amyloid *β*-Peptide

A major hallmark of AD is the overproduction of amyloid *β*-peptide (A*β*), which results in the formation of plaques. A*β* peptides are produced by successive proteolysis of amyloid precursor protein (APP) by *β*-site APP-cleaving enzyme-1 (BACE1) followed by *γ*-secretase [7–9]. This cleavage is imprecise and produces A*β* variants, which include those ending at residues 40 (A*β*_40_) and 42 (A*β*_42_) [10]. The A*β*_42_ is deposited earliest and most abundantly in plaques [11]. A*β* has been shown to induce lipid peroxidation in brain cell membranes and increase production of the lipid peroxidation products 4-hydroxynonenal and acrolein, and this may in part account for neurodegeneration in AD brain [12].

Several groups have investigated the role of DHA-enriched diets in animal models of AD and amyloidosis. In a series of studies Hashimoto and colleagues pretreated rats with DHA (300 mg/kg per day for 12 weeks) before an infusion of A*β*_1-40_ [13–15]. DHA had a significantly protective effect against the decrease in learning ability and reduced the oxidative stress induced by the A*β* infusion in the cerebral cortex and hippocampus. Pretreatment with DHA also prevented A*β*-induced impairment of an avoidance ability-related memory function. This group also investigated the protective effects of the DHA pretreatment before A*β* infusion on synaptosomal membranes properties. DHA content significantly increased along with both lateral and rotational membrane fluidity, whereas the cholesterol to phospholipid molar ratio and lipid peroxidation decreased. 

Elevated cholesterol increases A*β* levels in both *in vitro* and *in vivo *models of AD, and the generation, accumulation, and clearance of A*β* are regulated by cholesterol [16–18], suggesting an important role for cholesterol in the pathogenesis of AD. Indeed, cholesterol is now a recognized risk factor in the pathogenesis of AD [18]. Furthermore, A*β* generation may be determined by dynamic interactions of APP with lipid rafts, since APP inside rafts undergoes cleavage by *β*-secretase, whereas APP outside rafts undergoes cleavage by *α*-secretase [19]. Lipid rafts are lateral assemblies of sphingolipids and cholesterol within the membrane [20], and it has been suggested that age-related increases in cholesterol in lipid rafts provides a cooperative environment for accumulation of A*β* in plasma membranes [21]. It is therefore of interest to note that omega-3 PUFAs reduce the level of cholesterol in neuronal membranes [22], moreover cholesterol has a low affinity for DHA-containing phospholipids and therefore alterations in the level of membrane DHA will affect the formation of lipid rafts [23, 24]. It may be that the effects of DHA on cholesterol levels and lipid raft formation represent an important, but as-yet relatively unexplored, neuroprotective mechanism. 

Recent work by Green and coworkers has shown that DHA reduces the levels of soluble and intraneuronal A*β* and somatodendritic tau protein in the 3xTg AD mouse model [25]. The reduction was attributed to a decrease in the steady-state levels of presenilin 1. Importantly, when DHA was combined with either arachidonic or docosapentaenoic acids (both omega-6 PUFAs) the efficacy of DHA diminished over time, with the effects lost by 9 months. However, the additional presence of docosapentaenoic acid in the diet reduced levels of early-stage phospho-tau epitopes, which correlated with the positive outcome of a reduction in phosphorylated (activated) c-Jun N-terminal kinase, a putative tau kinase. It may be that the interrelationship between omega-6 and omega-3 PUFAs is a further important but neglected area of research. DHA has also been shown to significantly increase levels of the sorting protein LR11/SorLA in primary rat neurons, aged nontransgenic mice, and aged DHA-depleted APPsw AD mice [26]. This increase reduces the trafficking of the amyloid precursor protein to secretases involved in the *β*-amyloidogenic pathway, and reduced LR11/SorLA expression is strongly correlated with AD neuropathology [27].

DHA also attenuates A*β* secretion in cytokine-stressed human neural cells, and this is accompanied by formation of neuroprotectin D1 [28]. 10,17S-docosatriene, also known as neuroprotectin D1, is a metabolite of DHA, which has been shown to have potent anti-inflammatory and neuroprotective effects in neural systems [29] and stroke [30]. DHA and neuroprotectin D1 were reduced in the hippocampus cornu ammonis region 1 (CA1) of AD brains, but not in the thalamus or occipital lobes of the same brains. Furthermore, expression of cytosolic phospholipase A_2_ and 15-lipoxygenase, which are key enzymes in neuroprotectin D1 biosynthesis, was altered in AD hippocampus. Neuroprotectin D1 also repressed the A*β*_42_-triggered activation of proinflammatory genes and upregulated the antiapoptotic genes encoding Bcl-2, Bcl-xl, and Bfl-1(A1). The soluble amyloid precursor protein-alpha (APP-*α*) stimulated the biosynthesis of neuroprotectin D1 from DHA. These results suggest that the beneficial effects of DHA may in part also be mediated via the production of neuroprotectin D1, which induces anti-apoptotic and neuroprotective gene expression and consequently suppresses A*β*_42_-induced neurotoxicity.

Positive effects of DHA treatment have not however been universally reported and a recent study by Arendash and co-workers found that a high omega-3 PUFA diet provided no significant benefit in terms of decreasing the levels of soluble/insoluble hippocampal A*β* levels or improving cognitive performance in neither amyloid precursor protein (APP)-sw and PS1 double transgenic or wild-type mice [31]. The authors did find that higher cortical levels of omega-6 PUFA in both the transgenic and wild type mice were associated with impaired cognitive function, as measured by the radial arm water maze and Morris water maze tests. It may be that if DHA is acting via a reduction in the steady-state levels of presenilin 1, as suggested by Green and co-workers [25], the overexpression of presenilin 1 in the transgenic mouse model used in this study overwhelms the capacity of DHA, and therefore this model may not accurately reflect the potential effects in patients, especially those affected by sporadic AD. Furthermore, the omega-3 PUFA experimental diet contained high levels of EPA, which may have antagonised the effects of DHA (4.7% EPA and 5.7% DHA expressed as % total fat) by for example competing for enzymes in the neuroprotectin D1 biosynthetic pathway. 

An additional mechanism leading to a decrease in A*β* levels has been suggested by a study investigating the effect of omega-3 PUFA enrichment on gene expression in aged rats [32]. In this study, there was a 10-fold increase in transthyretin transcription following treatment, and since transthyretin is an A*β* protein scavenger [33], the authors concluded that the omega-3 PUFA-induced expression could potentially prevent amyloid aggregate formation. Indeed Serot and co-workers found an inverse relationship between transthyretin levels in cerebrospinal fluid and the severity of dementia in AD patients [34]. 

Several lines of evidence indicate that alterations in retinoid signalling lead to A*β* accumulation. For example, vitamin A deprivation results in deposition of A*β* in the cerebral blood vessels and downregulation of the retinoic acid receptor, RAR*α* in adult rat forebrain [35], and APP/presenilin 1 double mutant transgenic mice treated for 8 weeks with retinoic acid show significantly decreased A*β* deposition, tau phosphorylation, activation of microglia and astrocytes, attenuated neuronal degeneration and improved spatial memory compared to controls [36]. Retinoic acid regulates gene expression via its nuclear receptors: the retinoic acid receptors (RARs) and retinoid X receptors (RXRs) [37]. DHA and EPA have been reported to act as endogenous ligands of RXRs [38–40], and omega-3 PUFA supplementation has recently been shown to reverse age-related decreases in the levels of RAR*α*, RXR*α*, and RXR*β* in the aged rat forebrain [41]. It may be that omega-3 PUFAs are acting at a fundamental level of cell regulation by controlling gene expression via these receptors.

Omega-3 PUFA may also be acting at proliferator-activated receptors (PPARs), in particular PPAR*γ*. PPARs are involved in the control of the expression of a variety of genes involved in lipid energy metabolism and inflammation [42]. *In vitro* A*β* uptake and clearance from glial and neuronal medium is increased by PPAR*γ* [43]. In models of AD PPAR*γ* agonists reduce BACE1 transcription and expression in APP transgenic mice [44, 45], PPAR*γ* agonist and ibuprofen treatment reduce the expression of BACE1 and A*β*_42_ amyloid deposits in the hippocampus and cortex of APPV717I mice [46], and PPAR*γ* agonists protect neurons against A*β*-induced mitochondrial damage, apoptosis, and oxidative stress [47]. Furthermore, treatment with PPAR*γ* agonists significantly improves measures of cognition in mild AD and mild cognitively impaired subjects compared to a placebo [48]. PPAR*γ*'s are known to bind to and be activated by EPA, DHA, and DHA metabolites [49–51] and omega-3 PUFA supplementation has also been shown to reverse age-related decreases in the levels of PPAR*γ* in the aged rat forebrain [41].

There are a number of inflammatory events that occur in the brain as a response to the presence of A*β*. The key event appears to be the presence of activated microglia in the vicinity of the A*β*-containing plaques [52]. Microglial activation results in the sustained production of proinflammatory cytokines, growth factors, complement molecules, and adhesion molecules [53]. It has also been demonstrated that exposure of microglia to *β*-amyloid fibrils leads to the production of reactive oxygen species and neurotoxins [54] and activated microglia generate and release large numbers of superoxide ions [52], thereby increasing oxidative stress. The remainder of this paper will focus on this increased oxidative stress and inflammation and the potential role of omega-3 PUFA.

## 3. Oxidative Stress and Lipid Peroxidation

The importance of increased oxidative stress, lipid peroxidation, and lipid peroxidation products has consistently been shown in the pathogenesis of AD [4]. For example, increased lipid peroxidation has been detected in the frontal, temporal, parietal, and occipital cortices of AD patients [55]. Moreover, significantly increased levels of the lipid peroxidation products 4-hydroxynonenal and acrolein, are found in the hippocampus/parahippocampal gyrus, superior and middle temporal gyrus, and cerebellum of subjects with mild cognitive impairment and early AD compared to age-matched controls [56], suggesting that lipid peroxidation occurs as an early event in the pathogenesis of AD. This is consistent with evidence from animal models of AD, where increased oxidative stress is also found in the APPsw (Tg2576) transgenic mouse model of AD amyloidosis. Cerebral cortical and hippocampal homogenates were found to have higher levels of lipid peroxidation than those from wild-type mice, and lipid peroxidation preceded amyloid plaque formation [57]. 

Isoprostanes are prostaglandin-like compounds formed *in situ* by nonenzymatic free radical-catalysed lipid peroxidation [58]. F_2_-isoprostanes are derived from the omega-6 PUFA, arachidonic acid, F_3_-isoprostanes from EPA and F_4_-isoprostanes, also called neuroprostanes, from DHA [59–61]. Measurement of isoprostanes provides a sensitive marker of *in vivo* lipid peroxidation [62–64]. Measurements comparing the level of isoprostanes and neuroprostanes between AD and matched controls in different regions of postmortem brains show a PUFA-specific pattern of lipid peroxidation. There is an increase in esterified neuroprostanes in the occipital and temporal lobes, whereas F_2_-isoprostanes in these regions are unchanged [65]. Similar results were found by Reich and coworkers, in the superior and middle temporal gyri, hippocampus, inferior parietal lobule, and cerebral cortex [66]. There were increased levels of neuroprostanes while F_2_-isoprostanes levels were unchanged. These results show a selective pattern of lipid peroxidation occuring in AD, whereby DHA appears especially vulnerable and arachidonic acid is unaffected. 

These results may reflect more than simply the regional distribution of PUFA, since grey matter, where DHA is more abundant, has a significantly greater susceptibility to oxidative stress than white matter [64]. Levels of F_2_-isoprostanes and neuroprostanes was measured in the grey and white matter of brains from rats at intervals between 4 and 100 weeks of age. The level of neuroprostanes were consistently 20-fold greater than F_2_-isoprostanes, whereas DHA content was only two-fold greater than arachidonic acid, suggesting that DHA is especially prone to oxidative stress. 

PUFAs are particularly susceptible to lipid peroxidation and as such are a potential abundant source of destructive peroxidation products. The *in vitro* peroxidisability of unsaturated fatty acids is linearly dependent upon the number of bis-allylic positions, such that the peroxidisability of DHA is five times greater than that of the omega-6 PUFA linoleic acid, which contains only two double bonds [67]. This increased susceptibility would help to explain the vulnerability of membrane DHA to lipid peroxidation and may also suggest that elevating omega-3 PUFA intake *in situ*ations of increased oxidative stress, such as AD, would further increase production of toxic peroxidation products. 

Although some studies have shown increased lipid peroxidation following dietary supplementation with DHA and EPA in plasma, liver, and kidney [68–73] others have shown that EPA and DHA enriched diets do not increase levels of lipid peroxidation [74, 75]. Indeed, rather than increasing lipid peroxidation DHA has been shown to be neuroprotective by actually decreasing lipid peroxidation in the brain [76–78]. For example, rats being fed a diet enriched with DHA showed a significantly reduced level of cerebral lipid peroxide compared to controls [78], and the level of peroxidation was inversely related to the cerebral DHA/arachidonic acid ratio [77]. Furthermore, Tg2576 transgenic mice being fed a DHA deficient diet have significantly elevated levels of oxidised proteins compared to the control group, whereas the levels of oxidised proteins were significantly reduced in a DHA supplemented group [79]. 

Overall, these results suggest that DHA may function in an antioxidant role, at least in the brain. Yavin and colleagues have suggested a number of potential mechanisms for these observed antioxidant effects [80]. Membrane bound DHA may act as a trap for reactive oxygen species, DHA may be able to enhance the activity of endogenous antioxidant enzymes, or phosphatidylethanolamine plasmalogens containing DHA may contain intrinsic antioxidant properties. DHA has also been shown to induce antioxidant defences by enhancing cerebral activities of catalase, glutathione peroxidase, and levels of glutathione [81]. Furthermore, work by Green and colleagues suggests that DHA may reduce reactive oxygen species production by increasing nitric oxide production, which may decrease the cellular oxygen pool and consequently reduce the amount of reactive oxygen species and generate lipid peroxides [82].

## 4. Fatty Acid Composition

The increased lipid peroxidation and concomitant damage to membrane PUFA have the potential to significantly reduce the brain levels of PUFA and specifically DHA. However, a recent review found that low DHA is not consistently observed in plasma or brain of AD patients [83]. Although the authors did note wide variability between studies and suggested that it may be too early to draw any reliable conclusions. They did however report AD to be consistently associated with significantly lower phosphatidylethanolamine and phosphatidylcholine concentrations in the frontal cortex and hippocampus and the DHA content of hippocampal phosphatidylethanolamine is significantly lower compared to age-matched controls. 

DHA is particularly enriched in phosphatidylethanolamine [84], and this is therefore an important cellular store of DHA. Furthermore, in the hippocampal CA1 region of AD patients unesterified DHA and neuroprotectin D1 levels are reported to be about one-half and one-twentieth of those in age-matched controls, respectively [28]. It may therefore be hypothesised that although DHA decreases do not appear widespread or large in magnitude, these selective decreases in the hippocampus may have profound effects on specific cellular pools and the subsequent production of key DHA metabolites.

## 5. Inflammation

In comparison with nondemented elderly subjects virtually all the inflammatory cytokines and chemokines so far investigated appear to be upregulated in AD patients, including IL-1*β*, IL-6, TNF-*α*, IL-8, transforming growth factor-*β* (TGF-*β*), and macrophage inflammatory protein-1*α* (MIP-1*α*), for reviews see [3, 85]. The most notable proinflammatory cytokines produced by microglial cells, IL-1*α* and IL-1*β* are found throughout the brain of AD patients at autopsy [86]. IL-1*β* induces formation of reactive oxygen species, causing lipid peroxidation, and depletes membrane PUFA levels [87, 88]. IL-1*β* also triggers microglial activation and increases expression of amyloid precursor protein [89, 90]. Age-related increases in IL-1*β* are coupled with increased activity of MAPK, c-jun-N-terminal kinases (JNK, a stress activated MAP kinase), and p38 kinase and enhanced caspase-3 activity (an effector of apoptosis) [91]. MAP kinases mediate many specific cellular responses, including apoptosis and stress responses. 

EPA supplementation has been reported to decrease the production of proinflammatory cytokines in rodents. For example, EPA reversed the age-related increases in IL-1*β* concentration and activation of p38 and caspase-3 [92]. The EPA supplementation also restored age-related decreases in arachidonic acid and DHA concentrations and decreased reactive oxygen species accumulation. It has also recently been shown that IL-1*β* reduces acetylcholine release in rats, which correlates with memory deficits, and EPA supplementation prevents the effects of IL-1*β* and significantly improves memory [93]. Degeneration of cholinergic neurons is thought to contribute to cognitive impairments in AD [94]. 

EPA and DHA are the precursors of a diverse array of second messengers, called resolvins and docosanoids, with potent anti-inflammatory and proresolving actions [30, 95, 96]. Resolution of inflammation has traditionally been thought of as a passive process; however, recent evidence suggests that resolution is an active process involving the biosynthesis of local lipid derived mediators within the resolution phase, called resolvins [96]. The D class resolvins, derived from DHA block TNF-*α*-induced IL-1*β* transcripts in brain [95] and resolvin E1 (RvE1), produced from EPA, reduces IL-12 production via the ChemR23 receptor [97]. 10,17S-docosatriene, also known as neuroprotectin D1 is produced from DHA [30] and potently blocks the generation of both TNF-*α* and IFN-c by stimulated T cells [98]. Together, these data suggest that the omega-3 PUFA metabolites have the potential to decrease inflammation by decreasing inflammatory cytokine production.

Most human dietary supplementation studies investigating the effects of omega-3 PUFA on cytokine production have typically examined the effects of high EPA rather than DHA preparations [99–105]. EPA and DHA have differing effects on inflammatory response and they should not be considered as having mechanistic equivalence [106, 107]. Furthermore, EPA and DHA produce distinct metabolites, and it may be that DHA is the more potent immunological modulator via production of neuroprotectin D1. A recent trial supplemented 174 AD patients with a high DHA preparation (1.7 g DHA and 0.6 g EPA or placebo for 6 months) [108]. The DHA/EPA supplemented patients had significantly reduced IL-1*β*, and IL-6 release from lipopolysaccharide stimulated peripheral blood mononuclear cells compared to the placebo group, although TNF-*α*, IL-8, IL-10 and granulocyte colony-stimulating factor were not affected. 

The mechanism by which DHA and EPA are able to attenuate inflammatory processes has yet to be elucidated; however it is likely that these observed effects are mediated at the level of gene regulation by modifying the actions of transcription factors, with nuclear factor-kappa B (NF-*κ*B) a being potential candidate. NF-*κ*B has many diverse functions in the nervous system and regulates a large number of genes encoding many inflammatory cytokines, such as IL-1*β*, IL-6, INF-*α*, and MCP-1; however, it also regulates anti- and proapoptotic proteins including Bcl-2, Bcl-xl, Bcl-xs, Bax, cyclooygenase-2, and inducible nitric oxide synthase [109]. NF-*κ*B activation occurs following a signalling cascade initiated by external inflammatory stimuli and involves the phosphorylation and subsequent degradation of the I*κ*B inhibitory subunit, which allows NF-*κ*B translocation to the nucleus and the subsequent activation of expression of target genes [109]. 

The role of NF-*κ*B appears to be determined by cell type and timing of activation. For example, activation of NF-*κ*B in neurons associated with amyloid deposits is currently thought to be neuroprotective, whereas induction of NF-*κ*B in glia may be neurotoxic. For reviews see [109–111]. EPA or omega-3 PUFA supplemented media decreases lipopolysaccharide-induced activation of NF-*κ*B and TNF-*α* levels in cultured macrophages [112, 113] and monocytes [114], importantly, DHA suppresses IL-6 production and activation of NF-*κ*B in lipopolysaccharide/interferon-*γ* stimulated glial cells [115], suggesting omega-3 PUFAs may have direct effects on inflammatory cytokine production via effects on the NF-*κ*B signalling pathway. However, their site of action in this pathway and specific effects in neurons are yet to be determined.

## 6. Conclusions

Although there are strong correlations between low tissue levels of omega-3 PUFA and increased risk of AD, and low dietary intakes of omega-3 PUFA and cognitive decline and AD [116, 117], the results of dietary intervention studies have so far failed to live up to expectations raised by the preclinical and epidemiological studies. In the first study to look at the effects of omega-3 PUFA in AD, Yehuda and colleagues conducted a 4 week double-blind trial with 100 AD patients [118]. The supplemented group showed improvements in mood, cooperativity, short-term memory, appetite, sleep, and spatial orientation, whereas no improvements were seen in the placebo group. A subsequent pilot study investigated the effects of 500 mg EPA given twice daily for 12 weeks in patients with AD [119]. No differences were found and the authors concluded that EPA had no effects on cognition. A larger study in 174 patients found that administration of 1.7 g of DHA and 0.6 g of EPA per day for 6 months in patients with mild to moderate AD did not delay the rate of cognitive decline [120], although, positive effects were observed in a small subgroup of patients with very mild AD (MMSE >27 points). 

Therefore, current evidence appears to indicate that the beneficial effects of omega-3 PUFAs are more related to limiting the progression of cognitive decline, as most clinical trials have so far failed to demonstrate the efficacy of omega-3 PUFA treatment after the onset of AD symptoms. However, it should be noted that preclinical studies have focused on the effects of DHA and its metabolites, whereas clinical studies have investigated EPA, or may have used insufficient doses of DHA. In brain tissue, DHA-derived metabolites promote resolution and protect neural cells from neurodegeneration [121] and may therefore be the more important omega-3 PUFA in modulating the secretion of cytokines and inhibiting neuroinflammation and oxidative stress in AD. Furthermore, recent evidence suggests that the beneficial effects of omega-3 PUFA on reducing the risk of dementia and AD may be reduced by the presence of the apolipoprotein E *ε*4 (*APOE* *ε*4) allele [122–124]. It is hoped that future clinical studies will help clarify these issues and the relative roles and utility of DHA and EPA in the treatment of AD.

## References

[1] Selkoe DJ (2001). Alzheimer’s disease: genes, proteins, and therapy. *Physiological Reviews*.

[2] Murphy MF, Sramek JJ, Kurtz NM, Carta A, Cutler NR (1998). *Alzheimer’s Disease: Optimizing the Development of the Next Generation of Therapeutic Compounds*.

[3] Akiyama H, Barger S, Barnum S, Bradt B, Bauer J, Cole GM, Cooper NR, Eikelenboom P, Emmerling M, Fiebich BL, Finch CE, Frautschy S, Griffin WST, Hampel H, Hull M, Landreth G, Lue L-F, Mrak R, MacKenzie IR, McGeer PL, O’Banion MK, Pachter J, Pasinetti G, Plata-Salaman C, Rogers J, Rydel R, Shen Y, Streit W, Strohmeyer R, Tooyoma I, Van Muiswinkel FL, Veerhuis R, Walker D, Webster S, Wegrzyniak B, Wenk G, Wyss-Coray T (2000). Inflammation and Alzheimer’s disease. *Neurobiology of Aging*.

[4] Markesbery WR (1997). Oxidative stress hypothesis in Alzheimer’s disease. *Free Radical Biology and Medicine*.

[5] Calder PC (2009). Polyunsaturated fatty acids and inflammatory processes: new twists in an old tale. *Biochimie*.

[6] Dyall SC, Michael-Titus AT (2008). Neurological benefits of omega-3 fatty acids. *NeuroMolecular Medicine*.

[7] De Strooper B, Saftig P, Craessaerts K, Vanderstichele H, Guhde G, Annaert W, Von Figura K, Van Leuven F (1998). Deficiency of presenilin-1 inhibits the normal cleavage of amyloid precursor protein. *Nature*.

[8] Edbauer D, Winkler E, Regula JT, Pesold B, Steiner H, Haass C (2003). Reconstitution of *γ*-secretase activity. *Nature Cell Biology*.

[9] Vassar R, Bennett BD, Babu-Khan S, Kahn S, Mendiaz EA, Denis P, Teplow DB, Ross S, Amarante P, Loeloff R, Luo Y, Fisher S, Fuller J, Edenson S, Lile J, Jarosinski MA, Biere AL, Curran E, Burgess T, Louis J-C, Collins F, Treanor J, Rogers G, Citron M (1999). *β*-Secretase cleavage of Alzheimer’s amyloid precursor protein by the transmembrane aspartic protease BACE. *Science*.

[10] Cole SL, Vassar R (2008). The role of amyloid precursor protein processing by BACE1, the *β*-secretase, in Alzheimer disease pathophysiology. *Journal of Biological Chemistry*.

[11] Mann DMA, Iwatsubo T, Cairns NJ (1996). Amyloid *β* protein (A*β*) deposition in chromosome 14-linked Alzheimer’s disease: predominance of A*β*42(43). *Annals of Neurology*.

[12] Butterfield DA, Lauderback CM (2002). Lipid peroxidation and protein oxidation in Alzheimer’s disease brain: potential causes and consequences involving amyloid *β*-peptide-associated free radical oxidative stress. *Free Radical Biology and Medicine*.

[13] Hashimoto M, Hossain S, Shimada T, Sugioka K, Yamasaki H, Fuj Y, Ishibashi Y, Oka J-I, Shido O (2002). Docosahexaenoic acid provides protection from impairment of learning ability in Alzheimer’s disease model rats. *Journal of Neurochemistry*.

[14] Hashimoto M, Hossain S, Agdul H, Shido O (2005). Docosahexaenoic acid-induced amelioration on impairment of memory learning in amyloid *β*-infused rats relates to the decreases of amyloid *β* and cholesterol levels in detergent-insoluble membrane fractions. *Biochimica et Biophysica Acta*.

[15] Hashimoto M, Hossain S, Shimada T, Shido O (2006). Docosahexaenoic acid-induced protective effect against impaired learning in amyloid *β*-infused rats is associated with increased synaptosomal membrane fluidity. *Clinical and Experimental Pharmacology and Physiology*.

[16] Simons M, Keller P, de Strooper B, Beyreuther K, Dotti CG, Simons K (1998). Cholesterol depletion inhibits the generation of *β*-amyloid in hippocampal neurons. *Proceedings of the National Academy of Sciences of the United States of America*.

[17] Wood WG, Schroeder F, Igbavboa U, Avdulov NA, Chochina SV (2002). Brain membrane cholesterol domains, aging and amyloid beta-peptides. *Neurobiology of Aging*.

[18] Puglielli L, Tanzi RE, Kovacs DM (2003). Alzheimer’s disease: the cholesterol connection. *Nature Neuroscience*.

[19] Ehehalt R, Keller P, Haass C, Thiele C, Simons K (2003). Amyloidogenic processing of the Alzheimer *β*-amyloid precursor protein depends on lipid rafts. *Journal of Cell Biology*.

[20] Simons K, Toomre D (2000). Lipid rafts and signal transduction. *Nature Reviews Molecular Cell Biology*.

[21] Wood WG, Schroeder F, Igbavboa U, Avdulov NA, Chochina SV (2002). Brain membrane cholesterol domains, aging and amyloid beta-peptides. *Neurobiology of Aging*.

[22] Yehuda S, Rabinovitz S, Carasso RL, Mostofsky DI (2002). The role of polyunsaturated fatty acids in restoring the aging neuronal membrane. *Neurobiology of Aging*.

[23] Shaikh SR, Brzustowicz MR, Gustafson N, Stillwell W, Wassall SR (2002). Monounsaturated PE does not phase-separate from the lipid raft molecules sphingomyelin and cholesterol: role for polyunsaturation?. *Biochemistry*.

[24] Shaikh SR, Cherezov V, Caffrey M, Stillwell W, Wassall SR (2003). Interaction of cholesterol with a docosahexaenoic acid-containing phosphatidylethanolamine: trigger for microdomain/raft formation?. *Biochemistry*.

[25] Green KN, Martinez-Coria H, Khashwji H, Hall EB, Yurko-Mauro KA, Ellis L, LaFerla FM (2007). Dietary docosahexaenoic acid and docosapentaenoic acid ameliorate amyloid-*β* and tau pathology via a mechanism involving presenilin 1 levels. *Journal of Neuroscience*.

[26] Ma Q-L, Teter B, Ubeda OJ, Morihara T, Dhoot D, Nyby MD, Tuck ML, Frautschy SA, Cole GM (2007). Omega-3 fatty acid docosahexaenoic acid increases SorLA/LR11, a sorting protein with reduced expression in sporadic Alzheimer’s disease (AD): relevance to AD prevention. *Journal of Neuroscience*.

[27] Offe K, Dodson SE, Shoemaker JT, Fritz JJ, Gearing M, Levey AI, Lah JJ (2006). The lipoprotein receptor LR11 regulates amyloid *β* production and amyloid precursor protein traffic in endosomal compartments. *Journal of Neuroscience*.

[28] Lukiw WJ, Cui J-G, Marcheselli VL, Bodker M, Botkjaer A, Gotlinger K, Serhan CN, Bazan NG (2005). A role for docosahexaenoic acid-derived neuroprotectin D1 in neural cell survival and Alzheimer disease. *Journal of Clinical Investigation*.

[29] Mukherjee PK, Marcheselli VL, Serhan CN, Bazan NG (2004). Neuroprotectin D1: a docosahexaenoic acid-derived docosatriene protects human retinal pigment epithelial cells from oxidative stress. *Proceedings of the National Academy of Sciences of the United States of America*.

[30] Marcheselli VL, Hong S, Lukiw WJ, Tian XH, Gronert K, Musto A, Hardy M, Gimenez JM, Chiang N, Serhan CN, Bazan NG (2003). Novel docosanoids inhibit brain ischemia-reperfusion-mediated leukocyte infiltration and pro-inflammatory gene expression. *Journal of Biological Chemistry*.

[31] Arendash GW, Jensen MT, Salem N, Hussein N, Cracchiolo J, Dickson A, Leighty R, Potter H (2007). A diet high in omega-3 fatty acids does not improve or protect cognitive performance in Alzheimer’s transgenic mice. *Neuroscience*.

[32] Barceló-Coblijn G, Kitajka K, Puskás LG, Hogyes E, Zvara A, Hackler L, Farkas T (2003). Gene expression and molecular composition of phospholipids in rat brain in relation to dietary n-6 to n-3 fatty acid ratio. *Biochimica et Biophysica Acta *.

[33] Link CD (1995). Expression of human *β*-amyloid peptide in transgenic *Caenorhabditis elegans*. *Proceedings of the National Academy of Sciences of the United States of America*.

[34] Serot J-M, Christmann D, Dubost T, Couturier M (1997). Cerebrospinal fluid transthyretin: aging and late onset Alzheimer’s disease. *Journal of Neurology Neurosurgery and Psychiatry*.

[35] Corcoran JPT, So PL, Maden M (2004). Disruption of the retinoid signalling pathway causes a deposition of amyloid *β* in the adult rat brain. *European Journal of Neuroscience*.

[36] Ding Y, Qiao A, Wang Z, Goodwin JS, Lee E-S, Block ML, Allsbrook M, McDonald MP, Fan G-H (2008). Retinoic acid attenuates *β*-amyloid deposition and rescues memory deficits in an Alzheimer’s disease transgenic mouse model. *Journal of Neuroscience*.

[37] Mangelsdorf DJ, Evans DA (1995). The RXR heterodimers and orphan receptors. *Cell*.

[38] de Urquiza AM, Liu S, Sjoberg M, Zetterstrom RH, Griffiths W, Sjovall J, Perlmann T (2000). Docosahexaenoic acid, a ligand for the retinoid X receptor in mouse brain. *Science*.

[39] Egea PF, Mitschler A, Moras D (2002). Molecular recognition of agonist ligands by RXRs. *Molecular Endocrinology*.

[40] Lengqvist J, Mata de Urquiza A, Bergman A-C, Willson TM, Sjövall J, Perlmann T, Griffiths WJ (2004). Polyunsaturated fatty acids including docosahexaenoic and arachidonic acid bind to the retinoid X receptor *α* ligand-binding domain. *Molecular and Cellular Proteomics*.

[41] Dyall SC, Michael GJ, Michael-Titus AT (2010). Omega-3 fatty acids reverse age-related decreases in nuclear receptors and increase neurogenesis in old rats. *Journal of Neuroscience Research*.

[42] Desvergne B, Wahli W (1999). Peroxisome proliferator-activated receptors: nuclear control of metabolism. *Endocrine Reviews*.

[43] Camacho IE, Serneels L, Spittaels K, Merchiers P, Dominguez D, De Strooper B (2004). Peroxisome proliferator-activated receptor *γ* induces a clearance mechanism for the amyloid-*β* peptide. *Journal of Neuroscience*.

[44] Sastre M, Dewachter I, Landreth GE (2003). Nonsteroidal anti-inflammatory drugs and peroxisome proliferator-activated receptor-gamma agonists modulate immunostimulated processing of amyloid precursor protein through regulation of beta-secretase. *Journal of Neuroscience*.

[45] Sastre M, Dewachter I, Rossner S (2006). Nonsteroidal anti-inflammatory drugs repress *β*-secretase gene promoter activity by the activation of PPAR*γ*. *Proceedings of the National Academy of Sciences of the United States of America*.

[46] Heneka MT, Sastre M, Dumitrescu-Ozimek L (2005). Acute treatment with the PPARgamma agonist pioglitazone and ibuprofen reduces glial inflammation and Abeta1-42 levels in APPV717I transgenic mice. *Brain*.

[47] Fuenzalida K, Quintanilla R, Ramos P, Piderit D, Fuentealba RA, Martinez G, Inestrosa NC, Bronfman M (2007). Peroxisome proliferator-activated receptor *γ* up-regulates the Bcl-2 anti-apoptotic protein in neurons and induces mitochondrial stabilization and protection against oxidative stress and apoptosis. *Journal of Biological Chemistry*.

[48] Watson GS, Cholerton BA, Reger MA, Baker LD, Plymate SR, Asthana S, Fishel MA, Kulstad JJ, Green PS, Cook DG, Kahn SE, Keeling ML, Craft S (2005). Preserved cognition in patients with early Alzheimer disease and amnestic mild cognitive impairment during treatment with rosiglitazone: a preliminary study. *American Journal of Geriatric Psychiatry*.

[49] Chambrier C, Bastard J-P, Rieusset J, Chevillotte E, Bonnefont-Rousselot D, Therond P, Hainque B, Riou J-P, Laville M, Vidal H (2002). Eicosapentaenoic acid induces mRNA expression of peroxisome proliferator-activated receptor *γ*. *Obesity Research*.

[50] Itoh T, Murota I, Yoshikai K, Yamada S, Yamamoto K (2006). Synthesis of docosahexaenoic acid derivatives designed as novel PPAR*γ* agonists and antidiabetic agents. *Bioorganic and Medicinal Chemistry Letters*.

[51] Yamamoto K, Itoh T, Abe D, Shimizu M, Kanda T, Koyama T, Nishikawa M, Tamai T, Ooizumi H, Yamada S (2005). Identification of putative metabolites of docosahexaenoic acid as potent PPAR*γ* agonists and antidiabetic agents. *Bioorganic and Medicinal Chemistry Letters*.

[52] McGeer EG, McGeer PL (2003). Inflammatory processes in Alzheimer’s disease. *Progress in Neuro-Psychopharmacology and Biological Psychiatry*.

[53] Griffin WST (2006). Inflammation and neurodegenerative diseases. *American Journal of Clinical Nutrition*.

[54] Combs CK, Johnson DE, Cannady SB, Lehman TM, Landreth GE (1999). Identification of microglial signal transduction pathways mediating a neurotoxic response to amyloidogenic fragments of *β*-amyloid and prion proteins. *Journal of Neuroscience*.

[55] Miranda MD, de Bruin VMS, Vale MR, Viana GSB (2000). Lipid peroxidation and nitrite plus nitrate levels in brain tissue from patients with Alzheimer’s disease. *Gerontology*.

[56] Williams TI, Lynn BC, Markesbery WR, Lovell MA (2006). Increased levels of 4-hydroxynonenal and acrolein, neurotoxic markers of lipid peroxidation, in the brain in Mild Cognitive Impairment and early Alzheimer’s disease. *Neurobiology of Aging*.

[57] Praticò D, Uryu K, Leight S, Trojanoswki JQ, Lee VM-Y (2001). Increased lipid peroxidation precedes amyloid plaque formation in an animal model of alzheimer amyloidosis. *Journal of Neuroscience*.

[58] Morrow JD, Awad JA, Boss HJ, Blair IA, Roberts LJ (1992). Non-cyclooxygenase-derived prostanoids (F_2_-isoprostanes) are formed in situ on phospholipids. *Proceedings of the National Academy of Sciences of the United States of America*.

[59] Nourooz-Zadeh J, Halliwell B, Änggård EE (1997). Evidence for the formation of F_3_-isoprostanes during peroxidation of eicosapentaenoic acid. *Biochemical and Biophysical Research Communications*.

[60] Nourooz-Zadeh J, Liu EHC, Änggård EE, Halliwell B (1998). F_4_-isoprostanes: a novel class of prostanoids formed during peroxidation of docosahexaenoic acid (DHA). *Biochemical and Biophysical Research Communications*.

[61] Fam SS, Murphey LJ, Terry ES, Zackert WE, Chen Y, Gao L, Pandalai S, Milne GL, Jackson Roberts L, Porter NA, Montine TJ, Morrow JD (2002). Formation of highly reactive A-ring and J-ring isoprostane-like compounds (A4/J4-neuroprostanes) in vivo from docosahexaenoic acid. *Journal of Biological Chemistry*.

[62] Cracowski J-L, Durand T, Bessard G (2002). Isoprostanes as a biomarker of lipid peroxidation in humans: physiology, pharmacology and clinical implications. *Trends in Pharmacological Sciences*.

[63] Roberts LJ, Reckelhoff JF (2001). Measurement of F_2_-isoprostanes unveils profound oxidative stress in aged rats. *Biochemical and Biophysical Research Communications*.

[64] Youssef JA, Birnbaum LS, Swift LL, Morrow JD, Badr MZ (2003). Age-independent, gray matter-localized, brain-enhanced oxidative stress in male fischer 344 rats: brain levels of F2-isoprostanes and F4-neuroprostanes. *Free Radical Biology and Medicine*.

[65] Nourooz-Zadeh J, Liu EHC, Yhlen B, Änggård EE, Halliwell B (1999). F_4_-isoprostanes as specific marker of docosahexaenoic acid peroxidation in Alzheimer’s disease. *Journal of Neurochemistry*.

[66] Reich EE, Markesbery WR, Roberts LJ, Swift LL, Morrow JD, Montine TJ (2001). Brain regional quantification of F-ring and D-/E-ring isoprostanes and neuroprostanes in Alzheimer’s disease. *American Journal of Pathology*.

[67] Cosgrove JP, Church DF, Pryor WA (1987). The kinetics of the autoxidation of polyunsaturated fatty acids. *Lipids*.

[68] Kubo K, Saito M, Tadokoro T, Maekawa A (1997). Changes in susceptibility of tissues to lipid peroxidation after ingestion of various levels of docosahexaenoic acid and vitamin E. *British Journal of Nutrition*.

[69] Meydani M, Natiello F, Goldin B, Free N, Woods M, Schaefer E, Blumberg JB, Gorbach SL (1991). Effect of long-term fish oil supplementation on vitamin E status and lipid peroxidation in women. *Journal of Nutrition*.

[70] Jenkinson A, Franklin MF, Wahle K, Duthie GG (1999). Dietary intakes of polyunsaturated fatty acids and indices of oxidative stress in human volunteers. *European Journal of Clinical Nutrition*.

[71] Allard JP, Kurian R, Aghdassi E, Muggli R, Royall D (1997). Lipid peroxidation during n-3 fatty acid and vitamin E supplementation in humans. *Lipids*.

[72] Grundt H, Nilsen DWT, Mansoor MA, Nordøy A (2003). Increased lipid peroxidation during long-term intervention with high doses of n-3 fatty acids (PUFAs) following an acute myocardial infarction. *European Journal of Clinical Nutrition*.

[73] Song JH, Miyazawa T (2001). Enhanced level of n-3 fatty acid in membrane phospholipids induces lipid peroxidation in rats fed dietary docosahexaenoic acid oil. *Atherosclerosis*.

[74] Ando K, Nagata K, Yoshida R, Kikugawa K, Suzuki M (2000). Effect of n-3 polyunsaturated fatty acid supplementation on lipid peroxidation of rat organs. *Lipids*.

[75] Higdon JV, Liu J, Du S-H, Morrow JD, Ames BN, Wander RC (2000). Supplementation of postmenopausal women with fish oil rich in eicosapentaenoic acid and docosahexaenoic acid is not associated with greater in vivo lipid peroxidation compared with oils rich in oleate and linoleate as assessed by plasma malondialdehyde and F_2_-isoprostanes. *American Journal of Clinical Nutrition*.

[76] Green P, Glozman S, Weiner L, Yavin E (2001). Enhanced free radical scavenging and decreased lipid peroxidation in the rat fetal brain after treatment with ethyl docosahexaenoate. *Biochimica et Biophysica Acta*.

[77] Hossain MS, Hashimoto M, Masumura S (1998). Influence of docosahexaenoic acid on cerebral lipid peroxide level in aged rats with and without hypercholesterolemia. *Neuroscience Letters*.

[78] Kubo K, Saito M, Tadokoro T, Maekawa A (1998). Dietary docosahexaenoic acid dose not promote lipid peroxidation in rat tissue to the extent expected from peroxidizability index of the lipids. *Bioscience, Biotechnology and Biochemistry*.

[79] Lim GP, Calon F, Morihara T, Yang F, Teter B, Ubeda O, Salem N, Frautschy SA, Cole GM (2005). A diet enriched with the omega-3 fatty acid docosahexaenoic acid reduces amyloid burden in an aged Alzheimer mouse model. *Journal of Neuroscience*.

[80] Yavin E, Brand A, Green P (2002). Docosahexaenoic acid abundance in the brain: a biodevice to combat oxidative stress. *Nutritional Neuroscience*.

[81] Hossain MS, Hashimoto M, Gamoh S, Masumura S (1999). Antioxidative effects of docosahexaenoic acid in the cerebrum versus cerebellum and brainstem of aged hypercholesterolemic rats. *Journal of Neurochemistry*.

[82] Green P, Glozman S, Yavin E (2001). Ethyl docosahexaenoate-associated decrease in fetal brain lipid peroxide production is mediated by activation of prostanoid and nitric oxide pathways. *Biochimica et Biophysica Acta*.

[83] Plourde M, Fortier M, Vandal M, Tremblay-Mercier J, Freemantle E, Bégin M, Pifferi F, Cunnane SC (2007). Unresolved issues in the link between docosahexaenoic acid and Alzheimer’s disease. *Prostaglandins Leukotrienes and Essential Fatty Acids*.

[84] Dyall SC, Michael GJ, Whelpton R, Scott AG, Michael-Titus AT (2007). Dietary enrichment with omega-3 polyunsaturated fatty acids reverses age-related decreases in the GluR2 and NR2B glutamate receptor subunits in rat forebrain. *Neurobiology of Aging*.

[85] Wyss-Coray T (2006). Inflammation in Alzheimer disease: driving force, bystander or beneficial response?. *Nature Medicine*.

[86] Griffin WST, Stanley LC, Ling C, White L, MacLeod V, Perrot LJ, White CL, Araoz C (1989). Brain interleukin 1 and S-100 immunoreactivity are elevated in Down syndrome and Alzheimer disease. *Proceedings of the National Academy of Sciences of the United States of America*.

[87] Murray CA, Lynch MA (1998). Evidence that increased hippocampal expression of the cytokine interleukin-1*β* is a common trigger for age- and stress-induced impairments in long-term potentiation. *Journal of Neuroscience*.

[88] Murray CA, Clements MP, Lynch MA (1999). Interleukin-1 induces lipid peroxidation and membrane changes in rat hippocampus: an age-related study. *Gerontology*.

[89] Godoy MCP, Tarelli R, Ferrari CC, Sarchi MI, Pitossi FJ (2008). Central and systemic IL-1 exacerbates neurodegeneration and motor symptoms in a model of Parkinson’s disease. *Brain*.

[90] Griffin WST, Liu L, Li Y, Mrak RE, Barger SW (2006). Interleukin-1 mediates Alzheimer and Lewy body pathologies. *Journal of Neuroinflammation*.

[91] Lynch AM, Lynch MA (2002). The age-related increase in IL-1 type I receptor in rat hippocampus is coupled with an increase in caspase-3 activation. *European Journal of Neuroscience*.

[92] Martin DSD, Lonergan PE, Boland B, Fogarty MP, Brady M, Horrobin DF, Campbell VA, Lynch MA (2002). Apoptotic changes in the aged brain are triggered by interleukin-1*β*-induced activation of p38 and reversed by treatment with eicosapentaenoic acid. *Journal of Biological Chemistry*.

[93] Taepavarapruk P, Song C (2010). Reductions of acetylcholine release and nerve growth factor expression are correlated with memory impairment induced by interleukin-1*β* administrations: effects of omega-3 fatty acid EPA treatment. *Journal of Neurochemistry*.

[94] Coulson EJ, May LM, Sykes AM, Hamlin AS (2009). The role of the p75 neurotrophin receptor in cholinergic dysfunction in Alzheimer’s disease. *Neuroscientist*.

[95] Hong S, Gronert K, Devchand PR, Moussignac R-L, Serhan CN (2003). Novel docosatrienes and 17S-resolvins generated from docosahexaenoic acid in murine brain, human blood, and glial cells: autacoids in anti-inflammation. *Journal of Biological Chemistry*.

[96] Serhan CN, Chiang N (2008). Endogenous pro-resolving and anti-inflammatory lipid mediators: a new pharmacologic genus. *British Journal of Pharmacology*.

[97] Arita M, Bianchini F, Aliberti J, Sher A, Chiang N, Hong S, Yang R, Petasis NA, Serhan CN (2005). Stereochemical assignment, antiinflammatory properties, and receptor for the omega-3 lipid mediator resolvin E1. *Journal of Experimental Medicine*.

[98] Ariel A, Li P-L, Wang W, Tang W-X, Fredman G, Hong S, Gotlinger KH, Serhan CN (2005). The docosatriene protectin D1 is produced by TH2 skewing promotes human T cell via lipid raft clustering. *Journal of Biological Chemistry*.

[99] Billiar TR, Bankey PE, Svingen BA, Curran RD, West MA, Holman RT, Simmons RL, Cerra FB (1988). Fatty acid intake and Kupffer cell function: fish oil alters eicosanoid and monokine production to endotoxin stimulation. *Surgery*.

[100] Cooper AL, Gibbons L, Horan MA, Little RA, Rothwell NJ (1993). Effect of dietary fish oil supplementation on fever and cytokine production in human volunteers. *Clinical Nutrition*.

[101] Endres S, Ghorbani R, Kelley VE, Georgilis K, Lonnemann G, Van Der Meer JWM, Cannon JG, Rogers TS, Klempner MS, Weber PC, Schaefer EJ, Wolff SM, Dinarello CA (1989). The effect of dietary supplementation with n-3 polyunsaturated fatty acids on the synthesis of interleukin-1 and tumor necrosis factor by mononuclear cells. *New England Journal of Medicine*.

[102] Fritsche K (2006). Fatty acids as modulators of the immune response. *Annual Review of Nutrition*.

[103] Meydani SN, Endres S, Woods MM, Goldin BR, Soo C, Morrill-Labrode A, Dinarello CA, Gorbach SL (1991). Oral (n-3) fatty acid supplementation suppresses cytokine production and lymphocyte proliferation: comparison between young and older women. *Journal of Nutrition*.

[104] Meydani SN, Lichtenstein AH, Cornwall S, Meydani M, Goldin BR, Rasmussen H, Dinarello CA, Schaefer EJ (1993). Immunologic effects of national cholesterol education panel step-2 diets with and without fish-derived N-3 fatty acid enrichment. *Journal of Clinical Investigation*.

[105] Renier G, Skamene E, DeSanctis J, Radzioch D (1993). Dietary n-3 polyunsaturated fatty acids prevent the development of atherosclerotic lesions in mice. Modulation of macrophage secretory activities. *Arteriosclerosis and Thrombosis*.

[106] Kew S, Mesa MD, Tricon S, Bitckley R, Minihane AM, Yaqoob P (2004). Effects of oils rich in eicosapentaenoic and docosahexaenoic acids on immune cell composition and function in healthy humans. *American Journal of Clinical Nutrition*.

[107] Obajimi O, Black KD, MacDonald DJ, Boyle RM, Glen I, Ross BM (2005). Differential effects of eicosapentaenoic and docosahexaenoic acids upon oxidant-stimulated release and uptake of arachidonic acid in human lymphoma U937 cells. *Pharmacological Research*.

[108] Vedin I, Cederholm T, Levi YF (2008). Effects of docosahexaenoic acid-rich n-3 fatty acid supplementation on cytokine release from blood mononuclear leukocytes: the OmegAD study. *American Journal of Clinical Nutrition*.

[109] Malek R, Borowicz KK, Jargiello M, Czuczwar SJ (2007). Role of nuclear factor kappaB in the central nervous system. *Pharmacological Reports*.

[110] Kaltschmidt B, Kaltschmidt C (2009). NF-kappaB in the nervous system. *Cold Spring Harbor Perspectives in Biology*.

[111] Mattson MP, Camandola S (2001). NF-*κ*B in neuronal plasticity and neurodegenerative disorders. *Journal of Clinical Investigation*.

[112] Novak TE, Babcock TA, Jho DH, Helton WS, Espat NJ (2003). NF-*κ*B inhibition by *ω*-3 fatty acids modulates LPS-stimulated macrophage TNF-*α*-transcription. *American Journal of Physiology*.

[113] Lo C-J, Chiu KC, Fu M, Lo R, Helton S (1999). Fish oil decreases macrophage tumor necrosis factor gene transcription by altering the NF*κ*B activity. *Journal of Surgical Research*.

[114] Zhao Y, Joshi-Barve S, Barve S, Chen LH (2004). Eicosapentaenoic acid Prevents LPS-induced TNF-*α* expression by preventing NF-*κ*B activation. *Journal of the American College of Nutrition*.

[115] Pan H-C, Kao T-K, Ou Y-C, Yang D-Y, Yen Y-J, Wang C-C, Chuang Y-H, Liao S-L, Raung S-L, Wu C-W, Chiang A-N, Chen C-J (2009). Protective effect of docosahexaenoic acid against brain injury in ischemic rats. *Journal of Nutritional Biochemistry*.

[116] Barberger-Gateau P, Letenneur L, Deschamps V, Pérès K, Dartigues J-F, Renaud S (2002). Fish, meat, and risk of dementia: cohort study. *British Medical Journal*.

[117] Morris MC (2006). Docosahexaenoic acid and Alzheimer disease. *Archives of Neurology*.

[118] Yehuda S, Rabinovtz S, Carasso RL, Mostofsky DI (1996). Essential fatty acids preparation (SR-3) improves Alzheimer’s patients quality of life. *International Journal of Neuroscience*.

[119] Boston PF, Bennett A, Horrobin DF, Bennett CN (2004). Ethyl-EPA in Alzheimer’s disease—a pilot study. *Prostaglandins Leukotrienes and Essential Fatty Acids*.

[120] Freund-Levi Y, Eriksdotter-Jönhagen M, Cederholm T, Basun H, Faxén-Irving G, Garlind A, Vedin I, Vessby B, Wahlund L-O, Palmblad J (2006). *ω*-3 fatty acid treatment in 174 patients with mild to moderate Alzheimer disease: omegAD study: a randomized double-blind trial. *Archives of Neurology*.

[121] Bazan NG (2005). Neuroprotectin D1 (NPD1): a DHA-derived mediator that protects brain and retina against cell injury-induced oxidative stress. *Brain Pathology*.

[122] Huang TL, Zandi PP, Tucker KL (2005). Benefits of fatty fish on dementia risk are stronger for those without APOE epsilon4. *Neurology*.

[123] Plourde M, Vohl MC, Vandal M, Couture P, Lemieux S, Cunnane SC (2009). Plasma n-3 fatty acid response to an n-3 fatty acid supplement is modulated by apoE epsilon4 but not by the common PPAR-alpha L162V polymorphism in men. *British Journal of Nutrition*.

[124] Whalley LJ, Deary IJ, Starr JM, Wahle KW, Rance KA, Bourne VJ, Fox HC (2008). n-3 Fatty acid erythrocyte membrane content, APOE *ε*4, and cognitive variation: an observational follow-up study in late adulthood. *American Journal of Clinical Nutrition*.

